# Knowledge coordination in a semi-closed IM network under time-critical conditions: Making distributed expertise actionable

**DOI:** 10.1371/journal.pone.0359728

**Published:** 2026-09-30

**Authors:** Chih-Hsiung Chang, Jie Chi Yang

**Affiliations:** 1 Graduate Institute of Network Learning Technology, National Central University, Taiwan; 2 Department of Radio and Television, National Taiwan University of Arts, Taiwan; 3 Research Center for Science and Technology for Learning, National Central University, Taiwan; Kharkiv National Automobile and Highway University, UKRAINE

## Abstract

Semi-closed, cross-organizational instant messaging (IM) environments combine bounded membership with rapid communication across organizational boundaries. Building on research showing that expertise is distributed across professional networks, this study examines how journalists describe the coordination through which dispersed knowledge becomes collectively usable under time-critical conditions. A cross-organizational LINE group comprising Taiwanese television political journalists provides a revelatory case of digitally mediated epistemic coordination. Drawing on qualitative interviews with twelve participants across two experience cohorts, the study analytically reconstructs three interrelated mechanisms from recurrent patterns in participants’ accounts: the situational redistribution of epistemic authority, the externalization of tacit reasoning into interpretable cues, and the distributed assembly of interpretive support. The first author’s long-term insider experience served as interpretive context rather than independent evidence. Together, these mechanisms characterize digitally mediated epistemic coordination in the focal case: across participants’ accounts, distributed expertise became visible, evaluable, and actionable under time pressure. Participants also retrospectively linked repeated participation in such coordination to shifts in professional practice, describing sustained exposure to peers’ reasoning and evaluative criteria as contributing to the recalibration of their interpretive orientations. Taken together, the findings portray the focal LINE group as a consequential infrastructure for professional knowledge coordination under time-critical conditions.

## 1. Introduction

### 1.1. Knowledge coordination and the research problem

Knowledge workers in high-pressure, time-critical settings increasingly rely on mobile instant messaging (IM) to coordinate action, share expertise, and make sense of rapidly unfolding situations. Mobile IM platforms have emerged as everyday infrastructures for real-time professional coordination, enabling boundary-spanning interaction that operates independently of physical co-presence [1–3]. This study focuses on how professionals use semi-closed IM exchanges to coordinate distributed expertise in settings where membership is bounded, interaction is rapid, and coordination cannot rely on formal role assignment.

This study examines a single, long-standing cross-organizational LINE group of Taiwanese television political journalists as a revelatory case of digitally mediated epistemic coordination. Journalism provides an analytically productive context: reporters face extreme time pressure and cover rapidly evolving stories [4], while complex reporting requires them to draw on expertise distributed across organizations, beats, and networks [5]—conditions that intensify the need for rapid, distributed coordination [1,2]. The focal group was semi-closed and operated independently of any single newsroom, providing a bounded setting for examining how distributed expertise was coordinated across organizational boundaries. Recent research has examined professional IM groups in healthcare settings [6]; the present study extends this line of inquiry to cross-organizational knowledge coordination in time-critical journalism.

### 1.2. Distributed expertise, distributed cognition, and epistemic coordination

The study’s theoretical framework draws on distributed expertise and distributed cognition, while communication visibility and research on media affordances provide complementary lenses for specifying the mediated conditions of epistemic coordination. Distributed expertise [7] holds that expertise is dispersed across participants and becomes functional through collaborative activity rather than being concentrated in individuals. Research on communities of practice has examined how professional knowledge sharing can extend across organizational and practice boundaries and involve connecting and integrating different views [8]. In journalism, no individual reporter can possess all the domain knowledge and procedural or technical understanding relevant to a developing story, making access to knowledgeable insiders consequential [9].

Distributed cognition [10,11] extends this by shifting the unit of analysis from individual minds to socio-technical systems, showing that knowing and deciding can be properties of coordinated systems rather than isolated individuals. Together, these two frameworks establish that expertise and cognition are distributed and provide a conceptual basis for examining how professionals describe the mobilization and integration of dispersed knowledge in time-critical, conversational settings where coordination cannot rely solely on formally assigned roles [12–14].

Communication visibility scholarship complements these perspectives by explaining how computer-mediated communication can make others’ exchanges available for observation. Earlier affordance work conceptualized visibility as the possibility of making users’ behaviors, knowledge, preferences, and communication network connections more readily observable within organizations [15]. Leonardi [16] further showed how message transparency and network translucence can enhance metaknowledge of who knows what and who knows whom. He identified two behavioral changes through which workers developed and retained such metaknowledge for later use: a shift from experiential to vicarious learning through observation of others’ visible exchanges, and a shift from reactive search to proactive aggregation of metaknowledge. Treem et al. [17] subsequently conceptualized communication visibility as multidimensional, arising from communicators’ acts of making communication available, observers’ viewing, access, or exposure, and the sociomaterial context. Survey research has also linked perceived message transparency and network translucence with knowledge-brokering activity [18]. Together, this literature explains how digitally visible communication can make others’ knowledge and relationships more knowable.

More broadly, an affordance perspective distinguishes technical capabilities from the situated possibilities for action that emerge when users pursue particular purposes within specific social and organizational contexts. Media affordances are therefore relational rather than deterministic and may both enable and constrain organizational practice [19,20]. Research on team communication platforms has described the organization of group conversations into dedicated channels and flexible scaling across levels of synchronicity and media modality [21], while quantitative work on organizational IM has identified parallelism, transmission velocity, and symbol set among the media capabilities associated with adoption [3]. In the present study, this affordance-based distinction provides a vocabulary for interpreting participants’ accounts of rapid, multidirectional exchange that was less dependent on co-presence, an audience bounded by group membership, and the use of multiple representational formats, without treating these mediated conditions as automatically producing the three reconstructed mechanisms.

### 1.3. Research gap and study aims

Research on digital journalism has examined how journalists and news organizations use public-facing digital platforms and non-public messaging spaces to broaden their access to sources of news material [22], as well as how digital technologies reshape the production and validation of journalistic knowledge [23]. Building on these lines of research, the present study examines a related but more specific process: how participants described visible, partial contributions within the focal semi-closed, cross-organizational LINE group as being evaluated and assembled into time-sensitive, actionable professional judgment. The analysis is organized around three interrelated research questions:

**RQ1:** How do professionals in time-critical occupations use cross-organizational IM groups to coordinate practice and navigate rapidly evolving tasks?
**RQ2:** How do participants describe the coordination through which distributed expertise becomes visible, evaluable, and actionable within these digitally mediated environments?
**RQ3:** How does digitally mediated epistemic coordination relate to shifts in epistemic authority and professional practice in time-critical work, and what role does professional experience play in how participants contribute to and draw on this coordinative process?

## 2. Methodology

### 2.1. Research design

The focal professional setting for this study was a single, long-standing cross-organizational LINE group of Taiwanese television political journalists. All twelve interview participants were members of this group. Adopting an interview-based qualitative case study approach, the formal study used the twelve interview transcripts as its sole empirical dataset. The first author’s long-term professional participation and general research notes did not constitute a separate empirical dataset.

The first author, a television political journalist, was the only author who belonged to the focal LINE group. The first author joined the group when it was established in 2013 and continued participating through 2025. This insider position enabled access to the semi-closed professional setting and provided sustained familiarity with its context-specific language and shorthand, professional routines, and group norms. The first author conducted all twelve interviews and completed all coding and analysis.

The first author began doctoral study in 2015. From that point until the 2025 interviews, this ongoing participation supported contextual understanding but did not constitute systematic participant observation or a formally analyzed source of data. During this period, the first author maintained contemporaneous general research notes concerning recurring professional practices, group norms, context-specific terminology, and potentially researchable questions. These notes did not reproduce individual messages or identify particular message authors and were not coded. Formal empirical data collection for the study consisted of twelve interviews conducted in August 2025.

Insider familiarity also entailed risks of taking familiar practices for granted and interpreting the interview material through prior professional assumptions [24]. Consistent with Rind’s reflexive account of making prior assumptions explicit and reconsidering their influence on research decisions [25], the first author used the analytic memos to record developing interpretations that might have reflected prior professional assumptions. During theme development, the first author actively compared the provisional analysis with accounts that did not fit the broader pattern, narrowing or revising analytic claims when necessary. The audit trail documented negative cases and the reasons for revising or rejecting competing explanations. Long-term professional familiarity and the general research notes were used only to interpret professional terminology, situate participants’ accounts, and assess contextual plausibility. They did not serve as a basis for coding, theme development, or findings. All codes, themes, and findings were developed exclusively from the interview transcripts. Where prior expectations and the interview material diverged, interpretation followed the interview material.

### 2.2. Participants and data collection

#### 2.2.1. Sampling.

Purposive sampling was employed to recruit twelve Taiwanese television political journalists (n = 12) for semi-structured in-depth interviews. To examine the role of professional experience in epistemic coordination, participants were categorized into two broad groups (n = 6 per group) based on years of professional experience. Participants are referred to using cohort letters and numeric codes to protect confidentiality. All participants were recruited from 1 to 31 August 2025.

Group E (Experienced, n = 6): This group comprised experienced journalists. All participants had between 5 and 15 years of professional experience.

Group N (Novice, n = 6): This group consisted of early-career journalists. All participants had fewer than 5 years of professional experience.

The distinction between novice and experienced journalists was used as a broad analytical device rather than a rigid occupational classification. In this study, journalists with fewer than 5 years of experience were treated as novices because they were still in the relatively early stages of newsroom socialization and professional routine formation, whereas those with 5–15 years of experience were treated as experienced journalists because they had generally developed more stable reporting practices and source networks. Given the exploratory nature of qualitative research and the small sample size, this classification is intended to capture broad differences in professional experience and their potential bearing on how participants engage in knowledge coordination, rather than to establish fixed categories of expertise.

All participants were approached directly through the first author’s professional network, via face-to-face contact or instant messaging. All individuals invited to participate agreed to do so; no one declined or withdrew. All participants were aware of the first author’s dual role as both an academic researcher and a long-standing member of the same professional community. The adequacy of the sample was assessed in relation to the study’s focused aim, the specificity of the participant group, and the informational richness of the data, rather than statistical representativeness or population-level generalization [26].

#### 2.2.2. Interviews.

Each participant completed one semi-structured, face-to-face interview lasting approximately 1–2 hours. Interviews were audio-recorded and transcribed verbatim for analysis. The anonymized semi-structured interview guide, provided in English translation in S1 Appendix, covered three core domains: (1) professional socialization and knowledge acquisition, (2) technology-mediated coordination and collaborative practice, and (3) the role of IM environments in shaping professional cognition and coordination practices.

Although historical messages in the focal group remained technically accessible to the first author through group membership, no message histories were searched or revisited for research analysis. The study did not collect, export, code, or analyze LINE message histories, screenshots, timestamps, or message sequences, and no group message—whether produced by an interview participant or another group member—was treated as research data or used to corroborate interview accounts. Accordingly, the mechanisms reported below are analytic reconstructions of recurrent patterns in participants’ retrospective accounts rather than findings from direct digital interaction analysis.

### 2.3. Data Analysis

Data analysis was guided by the principles of Reflexive Thematic Analysis [27], following an iterative process of initial coding and thematic consolidation, progressing from initial meaning units to analytic categories and ultimately to core themes. Interview transcripts were coded through a concept-informed yet data-open approach. Concepts related to distributed expertise, epistemic authority, and role fluidity functioned as sensitizing concepts directing analytic attention, while themes were grounded in recurrent patterns in the data. The analysis proceeded in two steps: initial coding and theme consolidation were conducted first; concept-related interpretations were then developed during later rounds of consolidation.

Theme construction followed a systematic process: initial coding was open and organic, without a predetermined coding framework, and involved line-by-line engagement with each transcript to identify initial meaning units; related units were then grouped into analytic categories; finally, cross-category comparison was used to consolidate three core themes that formed the basis for the study’s analytically reconstructed mechanisms. Each core theme was developed from recurrent patterns across multiple interview accounts. The consolidation of higher-order analytic categories into core themes and analytically reconstructed mechanisms, together with participant coverage for each core theme, is presented in S2 Appendix.

Data adequacy was assessed across the full set of twelve interviews in relation to the study’s focused aim, the specificity of the participant group, and the informational richness and interpretive depth of the accounts [26]. Although experiential details varied, recurrent descriptions of coordination were identified across participants in both experience groups.

Data analysis, including coding and thematic consolidation, was conducted manually without the use of qualitative analysis software. To enhance rigor, interview accounts were compared across participants. During the 2025 analysis, a separate set of analytic memos documented coding decisions, theme consolidation, alternative interpretations, and broader analytical reflections. These memos formed part of the audit trail rather than an additional empirical dataset. Following the preliminary analysis, summaries of the three mechanisms were shared with six of the twelve participants for member reflection. They commented on whether the interpretations resonated with their professional experiences and identified points requiring clarification or revision.

### 2.4. Ethics, consent, and confidentiality

This study involved voluntary in-depth interviews with adult professionals. Under Article 4 of Taiwan’s Human Subjects Research Act, human subjects research is defined as research involving human specimens or information concerning individuals’ biological behavior, physiology, psychology, genetics, or medicine. An official interpretation issued by the then Department of Health (now the Ministry of Health and Welfare) on 22 March 2012 (Wei-Shu-Yi-Zi No. 1010064538) clarifies that this definition does not include social-behavioral research examining interactions between people and their social environments. Because the present qualitative interview study examined adult journalists’ professional interactions and practices, it fell within the social-behavioral category addressed by that interpretation. Accordingly, prior ethics committee review was not legally required under Taiwan’s current statutory framework, and the study was lawfully conducted without such review; no ethics approval or exemption number was applicable.

Before each interview, the first author explained the study’s purpose, the intended use of the data, participants’ right to withdraw at any time, and the measures used to protect confidentiality. All twelve participants provided written informed consent before participation and also provided verbal consent, documented in the audio recording, at the beginning of each interview. At the conclusion of each interview, participants were given an opportunity to clarify or withdraw any part of their responses before the transcript was finalized. Other members of the focal group were not recruited as research participants, and no consent was sought from them because no messages or other communications produced by them were collected or analyzed as research data. References by interviewees to colleagues or group coordination were treated solely as elements of the consenting interviewees’ retrospective accounts rather than as data obtained from those colleagues.

Interview quotations were attributed only to coded cohort-and-number identifiers (e.g., N1 and E3). Identifying details concerning participants, other individuals mentioned in the interviews, and relevant organizations were omitted or generalized in reporting. The manuscript does not reproduce any LINE screenshots, message text, or other message-level material. Because the focal professional community is small and closely interconnected, these procedures were designed to minimize, but cannot entirely eliminate, the risk of re-identification; reporting therefore followed a principle of minimal disclosure.

Audio recordings and transcripts were stored on a secure offline storage device without internet connectivity and were labeled only with coded participant identifiers. Access to the research materials was restricted, and only the first author retained access to the correspondence between the codes and participants’ identities. The interview data were used solely for academic purposes. Written consent forms and audio recordings will be securely deleted following the completion of all academic obligations associated with the study.

## 3. Findings

This section examines participants’ accounts of information access, interpretive coordination, and actionable judgment in time-critical journalistic work. Journalists described sharing updates, comparing interpretations, and coordinating next steps across organizational boundaries rather than relying solely on single-point guidance or physical co-presence. The analysis examines recurrent patterns in how dispersed knowledge became visible, evaluable, and actionable in everyday reporting practice.

### 3.1. Participation and information access in a semi-closed IM group

Participants described sharing assignment alerts, interviewee remarks, and on-site footage within the focal group. These information flows loosen the time–space constraints of point-to-point communication, enabling journalists who are not physically present to track developments and request materials as events unfold.

*My supervisor only tells me what topic they want, not who I should interview. Then, when they review the script, they suddenly say something is missing and tell me to fix it. So I have to look in the group to see who went and ask them to send me the footage. (N1, Novice)*

*Journalists covering the event post updates on the spot, ensuring the latest developments are always accessible. We can easily identify who is at the scene and reach out to them directly with any question. (N2, Novice)*

*Previously, when I called other journalists, I sometimes felt awkward or worried about disturbing them. But now, asking directly in a group chat doesn’t feel that way, because everyone in the group has similar real-time needs for their work. (E5, Experienced)*

*Due to limited manpower, it is not always possible to cover every assignment in person. Sometimes only a photojournalist is dispatched to a specific event, while I attend another location with a different photographer. However, I can monitor the progress of the other site through the group in real time, which is immensely helpful for my immediate reporting decisions and professional judgment. (E6, Experienced)*

Interview accounts indicate that junior members often contributed basic, low-threshold information.

*Whether it’s assignment notices or the transcript/key points of what interviewees said, we have to message our supervisors anyway. So sharing it in the group is basically effortless. (N3, Novice)*

*And since I’m still new, I don’t have many chances to ‘repay’ others. If I can contribute, I will. If someone asks me for footage, it’s fine—if I can help, I help. (N4, Novice)*

Senior journalists also emphasized the socialization function of reciprocity

*I don’t want others to think we don’t do anything and only ask for things. If you’re not recognized, that’s a problem. And helping others also lets you get to know people better and strengthens interaction with senior reporters. (E1, Experienced)*

Taken together, these accounts suggest that low-threshold contribution functions as an entry point into ongoing interaction. By contributing routine materials already produced in the course of their reporting work, junior members gain recognition, demonstrate reciprocity, and maintain access to the group’s information environment. What matters here is less a formalized membership role than a participation pattern through which access to distributed expertise becomes socially sustainable within a semi-closed network.

### 3.2. Peer support and real-time interpretive guidance

Participants described situations in which the news value of pooled information—including lengthy remarks or fragmented updates—was not immediately apparent to less-experienced journalists. In these accounts, more-experienced members provided concise summaries, emphasis cues, and contextual signals that made the information more interpretable and usable under time pressure.

*During interviews, we rookies often use our phones to write down everything because we’re afraid of missing something. We post it in the group. When I compare it with what senior reporters post, some seniors only write key points or the news point. Because we’re new and not that skilled yet, seniors’ concise notes actually show me what really matters—better than our verbatim transcripts. (N5, Novice)*

*Once a legislator talked for a long time. I wrote a lot but still couldn’t tell what the key was. Then a senior replied to my post and said, ‘This legislator finally loosened up and admitted something.’ After that explanation, I realized the key was hidden in just a few lines, and I finally understood the implied meaning. (N6, Novice)*

*Once, someone reported that a high-profile figure involved in a legal case had been transferred to a specific law enforcement agency, and everyone immediately prepared to head there for coverage. However, a senior reporter familiar with that regulatory beat intervened, reminding us that the individual was likely still under investigation at another unit and had not yet been transferred. Following this lead, the group redirected their efforts to the Bureau instead. (N1, Novice)*

This episode illustrates that interpretive support could involve correction as well as explanation: after a senior reporter with relevant beat-specific knowledge intervened, the group revised its initial assumption and redirected its reporting effort.

Some members also described actively turning group exchanges into materials for self-directed interpretation:

*When I was still new, even if it wasn’t a story I needed to do, I forced myself to read the transcripts in the group, analyze what the key points were, and then read the key-point discussions. That process sharpens my ability to interpret information and make reporting decisions. (E2, Experienced)*

Even for senior journalists, interpretation may require contextual, historical, and inferential support from others with relevant recall or beat-specific knowledge:

*Whether certain wording has hidden implications or historical background is something you have to keep developing a sense for. Seniors often remind everyone in the group that something that sounds ordinary is actually important—maybe there’s an implied message, or they’re making a move, or it connects to a past event. (E3, Experienced)*

*Some senior reporters also have great memory and pull up what the same person said before to question inconsistencies. Not every journalist would know that. The group gives us more chances to hear seniors’ sharing and guidance—they’re like a living database. (E4, Experienced)*

These accounts indicate that the group provides layered forms of interpretive support rather than uniform guidance.

*Sometimes I don’t quite understand it at first, but someone in the group will suggest a direction, then another person adds the background, and another brings up similar past cases. After seeing a few rounds of replies, the whole thing suddenly becomes clear. (N2, Novice)*

*It’s not that one person explains everything at once. Usually, one person drops a message, another adds the context, and someone else follows up with professional knowledge. Most of the time, it’s everyone piecing it together bit by bit that we finally know how to truly interpret it. (E5, Experienced)*

Some contributions help identify salient “news points,” while others supply contextual decoding, historical comparison, or inferential cues.

*Some senior journalists don’t just give a final conclusion when they reply; they’ll explicitly say, ‘The key point of this sentence is here,’ or ‘Why this statement is unusual.’ For us, it’s useful to see how they make those judgments. (N4, Novice)*

Taken together, these accounts illustrate how dispersed observations became usable interpretive signals and tacit professional judgment became available to other group members.

### 3.3. Situated sensemaking in ongoing reporting practice

News reporting is profoundly context-dependent. Interviewees portrayed the focal IM group as a shared environment for anticipatory discussion, real-time prompting, and collective interpretation before and during fieldwork. These exchanges were described as embedded in ongoing reporting practice rather than as separate from action, helping participants clarify angles, anticipate responses, and adjust next steps in context.

*Every morning people go through the day’s schedules in the group and share what each person will cover. We usually discuss who will show up, what the news focus might be, what questions to ask, and how to ask them… For novices or people unfamiliar with certain beats, it’s like real-time prompts. It’s not only helpful for work—it also helps us refine reporting judgments in real time. (E1, Experienced)*

*Once, I was about to rush to a specific location to get some footage, but someone in the group judged that another spot was more likely to have a story, so I changed my route. It turned out that their judgment was correct; that senior colleague indeed has a very deep understanding of those issues. (N6, Novice)*

*I often feel that the group is more powerful than AI. It’s not just that every question gets an answer; it lets you know what is happening and why, which directly helps with reporting strategies—such as where to go next, whom to find first, and which angle to prioritize. (E4, Experienced)*

This comparison is analytically significant: what this participant valued in peer IM interaction was not merely access to answers, but the capacity to situate information within unfolding contexts and translate it into actionable reporting strategy.

Across the accounts, these exchanges projected accumulated professional experience into a shared communication space, supporting immediate reporting needs while making evaluative reasoning available to other members.

*…A senior in the group warned that if we asked it directly, the person might walk away… I saw it with my own eyes and realized how well seniors understood this person’s personality. (N3, Novice)*

One participant described how an ill-defined request elicited multiple alternatives rather than a single answer:

*Once my supervisor wanted a feature on a highly specialized policy issue. I wasn’t familiar with it, so I asked in the group. People asked what angle I was going for and suggested possible entry points. They also gave me a lot of scholars’ contact numbers and explained what each person is good at and what they’re like. People didn’t totally agree—someone would say, ‘A is better on the diplomatic aspects,’ and someone else would go, ‘No, A is stronger on the technical details.’ After some back-and-forth, a few reporters who cover that specific beat helped me narrow it down and sent me the contact. After a bit more discussion, they said two scholars made more sense for my angle—because they follow defense strategy more closely and understand more of what goes on behind the scenes. One of them also had an office near our company, so he was the first choice. I reached out and he was free, so I went to see him. I got the interviewee, but I also came to understand who’s who in this field and how they decide who fits what. (E5, Experienced)*

In the participant’s account, coordination did not begin with full agreement but proceeded through the comparison of alternatives and the articulation of evaluative criteria. What circulated was therefore not only a contact, but also a shared set of comparative judgments that helped the participant understand how “fit” was assessed in practice.

Interviewees also recounted how photos, videos, and annotated representations shared by on-site members enabled off-site journalists to collaborate remotely and provide immediate interpretive support. In these accounts, such exchanges contributed to collective sensemaking around a shared situation.

*In early 2020, during the fatal military helicopter crash, since the Black Hawk was relatively new in Taiwan, the public didn’t really know the seat layout. But soon after the accident, a colleague who possessed specialized knowledge of the aircraft’s internal layout drew a seating diagram in the group. They explained the layout and how the military typically seats generals and subordinates. As casualty names came out, we used the likely seating positions to analyze possible impact conditions. Later, when the military released photos and videos, senior reporters combined the imagery with our earlier discussion to further analyze possible causes. Step by step, I came to better understand how to interpret aviation-related information in reporting. (E2, Experienced)*

Overall, participants portrayed the focal IM group as more than an information channel and as a setting for distributed interpretation, with reported exchanges combining factual information with orientation, comparison, and practical judgment.

### 3.4. Coordinating distributed expertise under time pressure

Participants’ retrospective accounts portrayed cross-organizational IM exchange during breaking news as supporting the coordination of distributed expertise under conditions of high complexity, information density, and severe time pressure. Drawing on Brown et al.’s [7] notion of distributed expertise, the analysis treats expertise not as a fixed possession of particular individuals but as a resource dispersed across participants that must be mobilized, aligned, and integrated to become usable for action.

Retrospective accounts of major incidents—including the 2016 Tainan earthquake and the 2019 Nanfang’ao bridge collapse—describe recurring coordination characterized by role fluidity and the use of multiple representational formats under rapidly evolving conditions. In both cases, the interviewees described temporary, event-specific groups they had joined outside the focal group. The first author was not a member of either group and did not access or observe these groups for research purposes; both cases entered the analysis solely through the interviewees’ retrospective accounts.

In 2016, a magnitude 6.4 earthquake known as the 0206 earthquake struck southern Taiwan two days before Lunar New Year, causing multiple building collapses and significant casualties.

*In 2016, the 0206 earthquake hit Tainan. Many buildings collapsed and people were trapped. It happened two days before the Lunar New Year, so many reporters were on leave. TV stations coordinated reporters from different regions to give up leave and support the coverage. When I arrived, the affected area was large and chaotic. Out-of-town reporters weren’t familiar with the city. I was on the politics beat and didn’t really understand the fire and rescue system. Even though the command center held briefings, they couldn’t update everything in real time. Luckily, colleagues on site created a group. If something happened in another area or someone was rescued, people shared it in the group. If you had questions, you could ask and get answers immediately. Reporters from different beats brought different experiences and expertise. Sometimes, if we didn’t understand a tool rescue workers were using, I recorded a short clip and posted it. A colleague familiar with rescue work replied: it was a life detector, used to locate survivors, and they explained the equipment and rescue process. A medical-beat reporter also explained emergency care and follow-up treatment. Another time, someone didn’t know which official was visiting the scene; they posted a photo, and I explained the person’s title. For us out-of-town reporters, this mutual help was hugely useful—and we gained a great deal of on-the-spot professional knowledge. (E3, Experienced)*

This case is consistent with a situational redistribution of epistemic authority: epistemic authority rested on the immediate relevance of participants’ situated expertise to the unfolding task rather than on seniority alone.

*I’ve noticed that in the group, being the most senior doesn’t necessarily mean you’re the most authoritative. It depends on who understands the specific issue being discussed; naturally, everyone will want to ask them and listen to their analysis. (N5, Novice)*

*When breaking news happens, the people who primarily provide intelligence and analysis are usually those familiar with that specific area. In those moments, seniority doesn’t matter at all; what matters is whether that person knows the most about the current development and the reasons behind the event. (E6, Experienced)*

In 2019, shortly after a typhoon had passed, a suspension bridge at Nanfang’ao fishing port in northeastern Taiwan suddenly collapsed, killing several fishermen aboard vessels beneath it.

*In 2019, there was a bridge collapse accident at Nanfang’ao fishing port in Yilan. A typhoon had just passed and the scene was chaotic. The bridge was broken, traffic was disrupted, and you had to detour to get in and out. Colleagues set up a group and added members—locals and reporters coming from other places. People shared on-site info. For example, some used photos to guide routes. At first we didn’t know where the rescue command was, so colleagues who arrived earlier took screenshots of Google Maps, drew routes, and posted photos to show where vehicles were restricted and where you could park, reminding everyone not to obstruct rescue work. Others shared rescue and search information, relayed what some interviewees said, and shared civil-engineering experts’ analysis of possible causes. People discussed the latest developments. Because a bridge collapse involves civil engineering, maintenance, and public safety, a colleague with an engineering background tried to synthesize possible reasons. The knowledge wasn’t easy, so others helped explain it. Through the information sharing and discussion, I gained some professional knowledge and also developed a better sense of how to approach interviewees on topics outside my own expertise. (E2, Experienced)*

In this account, members used map screenshots, route annotations, and photographs to translate fragmented spatial information into reporting routes, while external expert interpretations and peer explanations made specialized engineering reasoning more intelligible to non-specialists.

*Actually, it’s impossible for one person to know everything. For instance, some are more familiar with Congress, some with the ruling party, and others with the opposition parties. Normally, these are just small, separate pieces, but when you put them together in the group, a holistic judgment emerges. (E4, Experienced)*

*Most breaking news doesn’t rely on just one person; instead, everyone contributes the part they know best. After filtering or piecing together various information—what’s possible versus impossible, what’s correct versus incorrect—everyone knows the right way to cover the story next. (E6, Experienced)*

Taken together, participants’ accounts of these cases suggest that distributed expertise did not simply exist as a background condition but had to be coordinated to become usable. The analysis reconstructed three coordination mechanisms from recurrent patterns in these accounts: the situational redistribution of epistemic authority, the externalization of tacit reasoning into interpretable cues, and the distributed assembly of interpretive support. Together, these mechanisms capture how participants described such coordination in fast-moving reporting situations.

### 3.5. Episodic exchange and participants’ accounts of professional reorientation

Beyond immediate information exchange and task coordination, participants retrospectively described changes in reporting practices and interpretive assumptions.

*There was a time when a presidential candidate had about an hour of unexplained time between two public events. It felt like there might be a hidden itinerary. I asked in the group chat, and people started discussing possible explanations. Some reporters tried to ask the campaign staff, but they wouldn’t reveal anything. Eventually, a member in the group figured out that the candidate was secretly visiting supporters of the opposing camp to try to win them over. We went there to report the story, which really surprised the campaign staff. Later the member in the group explained that they had asked several local opinion leaders from different political parties whether the candidate planned to meet them. Someone hinted that the candidate would visit a particular person. Through that process I realized that you cannot rely only on official schedules released by politicians. Sometimes information is deliberately withheld. You need to look more broadly and explore the surrounding networks to find clues. I hope that next time I will be the one who discovers the lead and shares it with others. (N2, Novice)*

In this account, the novice journalist attributed a reassessment of the limits of official information channels to learning how another group member had pursued leads through local political networks.

Another participant described a related instance involving access to a retired official’s private contact information:

*Someone wanted to interview a high-ranking retired official but didn’t have his contact information. A senior reporter shared the phone number in the group but asked everyone not to circulate it. Actually, we all know this official because he often attends major events, but whenever we ask for his phone number he politely refuses. I asked the senior reporter how they obtained it. They said that when the official was still a junior staffer, they already thought he was promising, so they often talked with him about various issues and gradually built a personal relationship. That’s why they still have his private number today. The senior reporter first called to ask if the official would be willing to give an interview before sharing the contact. From that story I came to understand that building interview networks requires long-term vision. Since then I have paid more attention not only to immediate reporting tasks but also to the broader network around events and people. Maintaining relationships early on eventually becomes one’s real professional asset. (E1, Experienced)*

Access to the retired official’s private contact information was uneven and mediated through a senior reporter. By first confirming the official’s willingness to be interviewed and then sharing the number with an explicit request that it not be circulated further, the senior reporter extended access to other group members while protecting the long-standing source relationship on which that access depended.

*The group has made me different from when I first started. Now, when planning my interviews, I don’t just look at official information; I first think about who else nearby might know something and what other clues can be used for cross-verification. (N6, Novice)*

*I used to be very anxious about finding basic reporting information—those direct but superficial questions. But later, I started to consider whether there are other signals behind the news or if there were similar situations in the past. Actually, this was all influenced by the discussions in the group. (E3, Experienced)*

Across these accounts, participants retrospectively linked exposure to peers’ reasoning and evaluative criteria to shifts in their reporting routines and interpretive orientations.

## 4. Discussion

### 4.1. Boundary-spanning access in a semi-closed professional IM network

Addressing RQ1, participants’ accounts indicate that the focal cross-organizational LINE group restructured how participants accessed and coordinated distributed expertise under time pressure. In the situations participants described, peer reasoning and judgment became accessible across organizational boundaries, including to group members who were not physically present at the reporting site. The key shift in the focal case was not merely faster communication, but a change in how expertise access was organized—less location-bound and less dependent on one-to-one interpersonal requests. Participants portrayed the focal group not as a formal knowledge-sharing platform but as an ongoing communication infrastructure through which members maintained situational awareness and coordinated action across organizational boundaries.

Prior research on workplace IM documented how concurrent backstage exchanges restructured the temporal and socio-spatial boundaries of collaborative decision-making processes, including by allowing meeting participants to obtain just-in-time input from people outside the focal meeting [28]. In contrast, the present accounts concern a different communicative configuration in which task-relevant contributions circulated within a long-standing professional IM group that was not organized around a focal meeting. Using the affordance lens outlined in Section 1.2, the analysis highlights three features in participants’ descriptions of this configuration: rapid multidirectional exchange across organizational boundaries, a membership-defined audience, and the availability of text, photographs, videos, and annotated representations. Concepts from prior IM and platform research—including transmission velocity, parallelism, and symbol set [3], as well as group conversations organized into dedicated channels and flexible scaling across levels of synchronicity and media modality [21]—provide a comparative vocabulary for interpreting these descriptions. Because affordances are relational rather than deterministic and may enable as well as constrain action [19,20], the analysis does not treat the coordination described by participants as an inherent platform effect or as generated by these features alone. Rather, this vocabulary helps specify the mediated configuration in which participants described this shift in expertise access.

### 4.2. Three mechanisms of digitally mediated epistemic coordination

Addressing RQ2, the central contribution of this study is the analytic reconstruction of three interrelated mechanisms from recurrent patterns in participants’ retrospective accounts. These mechanisms specify how distributed expertise became actionable under time pressure in the focal semi-closed professional IM setting; the study does not claim to capture the turn-by-turn organization of message exchange.

***Mechanism 1: Situational redistribution of epistemic authority***

Across these accounts, epistemic authority shifts toward members whose task-specific knowledge is most relevant at a given moment rather than remaining anchored in organizational seniority. This mechanism captures the situational redistribution of epistemic authority according to task relevance under time pressure.

Accounts from both experience positions indicate that task-relevant contributions could flow in more than one direction: novice participants described contributing on-site information that more-experienced members might lack, while experienced participants described contributing contextual or historical knowledge that less-experienced members might not yet possess. Taken together, these accounts suggest that epistemic authority could shift across experience positions according to situational demands rather than seniority alone. Participants in both positions echoed this principle: one novice and one experienced participant each described task relevance as outweighing seniority in the allocation of epistemic authority. Another experienced participant’s earthquake account further illustrated role fluidity within a single episode: the participant drew on colleagues’ rescue and medical knowledge while contributing political-beat knowledge when a group member could not identify a visiting official. These accounts indicate that professional experience did not fix who contributed knowledge, who drew on others’ knowledge, or who held epistemic authority in a given situation.

***Mechanism 2: Externalization of tacit reasoning into interpretable cues***

Participants described how tacit professional judgment was expressed through concise summaries, contextual reminders, annotated representations, and evaluative comments. These cues conveyed not only information but also the criteria and reasoning that other members could consider and draw on in real time. This mechanism captures how otherwise tacit reasoning became more accessible, particularly to less-experienced participants.

***Mechanism 3: Distributed assembly of interpretive support***

Participants described interpretations as becoming progressively clearer through the partial and complementary contributions of multiple members rather than through centralized instruction. This mechanism captures the distributed assembly of those contributions into time-sensitive, actionable judgments.

The three mechanisms are analytically distinct but interrelated in explaining how participants described the coordination of distributed expertise under time pressure. They are not stages in a fixed sequence.

### Synthesis

Together, these mechanisms characterize digitally mediated epistemic coordination in the focal case: across participants’ accounts, distributed expertise became visible, evaluable, and actionable under time pressure. The three mechanisms were reconstructed within the focal case characterized by time-critical tasks, distributed expertise, multidirectional communication, bounded membership, and norms of reciprocity.

Participants’ accounts of reading exchanges beyond their immediate assignments and learning from peers’ visible reasoning are compatible with the third-party observation and vicarious learning described in communication visibility research, in which message transparency and network translucence can enhance metaknowledge of who knows what and who knows whom [16]. Treem et al.’s [17] multidimensional formulation further emphasizes that communication visibility depends on what communicators make available, what observers view, access, or become exposed to, and the sociomaterial context. The present analysis therefore does not treat visibility itself as either a fourth mechanism or the study’s distinctive contribution. Rather, it reconstructs how participants described situational epistemic authority shifting toward task-relevant contributors, tacit reasoning becoming available through interpretable cues, and partial contributions being assembled into time-sensitive, actionable professional judgment. The contribution lies in analytically specifying this reported coordination within the focal semi-closed, cross-organizational LINE group under acute time pressure; the study does not establish these contextual features as necessary or sufficient conditions for the mechanisms.

### 4.3. Ongoing reorientation of professional practice

Addressing RQ3, participants described shifts in epistemic authority and changes in professional practice associated with digitally mediated epistemic coordination, with the reported patterns differing across experience levels. Beyond immediate coordination, participants retrospectively attributed changes in their interpretive orientations and reporting strategies to sustained exposure to peers’ reasoning and evaluative criteria. The analysis treats these accounts as describing a reorientation of professional interpretation and practice rather than simply the accumulation of discrete knowledge. The contribution here is not to establish longitudinal change directly, but to show how participants understood repeated participation in coordination as contributing to the ongoing reorientation of professional interpretation and action.

Within these accounts, the reported reorientation differed across experience levels: novice members described developing more refined interpretive frameworks through sustained exposure to senior members’ reasoning. Experienced members, in turn, described recalibrating their own practice in response to the repeated need to articulate judgment under time pressure. These contrasting accounts suggest that the same coordinative environment can be associated with different forms of professional reorientation across experience positions without implying a single linear progression.

Participants’ accounts portray coordination and decision-making as closely intertwined in live reporting contexts: reported exchanges were described as contributing to the formation of decisions rather than occurring only after decisions had been made. Related research has identified mobile instant messaging as supporting collaborative problem solving and knowledge sharing in distributed networks of practice [29]. Participants also linked repeated exposure to peers’ reasoning with the reinterpretation of episodic interactions as practical guidance for future action.

Across these retrospective accounts, participants described developing not simply additional knowledge, but a more refined capacity to interpret signals, evaluate relevance, and act under uncertainty. Participants described continuing to learn from senior colleagues while also drawing on peers across organizational and beat boundaries. The analysis interprets these accounts as a repositioning rather than a displacement of traditional mentorship within a broader networked arrangement in which epistemic authority could extend beyond seniority and participants could draw on peers’ professional judgment across organizational boundaries.

Beyond journalism, these findings may offer a preliminary analytical lens for examining other knowledge-intensive professions characterized by time-critical decision-making, distributed expertise, and reliance on mobile communication infrastructures.

## 5. Conclusion

### 5.1. Summary of the study

Based on interviews with members of a focal cross-organizational LINE group, this study examined how distributed expertise became actionable under time-critical reporting conditions. From recurrent patterns in participants’ accounts, the analysis reconstructs three interrelated mechanisms: the situational redistribution of epistemic authority, the externalization of tacit reasoning into interpretable cues, and the distributed assembly of interpretive support.

Taken together, these mechanisms characterize distributed expertise as more than a structural condition of knowledge diversity: across participants’ accounts, it became visible, evaluable, and actionable under time pressure. Participants also retrospectively linked repeated participation in such coordination to shifts in professional practice, describing sustained exposure to peers’ reasoning and evaluative criteria as contributing to the recalibration of their interpretive orientations.

Within the focal LINE group, participants described drawing on and contributing task-relevant expertise across experience levels and organizational boundaries without relying solely on physical co-presence or dyadic mentorship. The analysis interprets this pattern as a repositioning rather than a displacement of traditional mentorship within a broader networked arrangement.

### 5.2. Limitations and future research

First, the study focuses on participants’ retrospective accounts of epistemic coordination in IM-based professional practice and does not systematically examine how such coordination relates to organizational governance, editorial oversight, or formal hierarchies within news organizations. Future research may examine how IM affordances intersect with institutional structures and managerial coordination.

Second, participants reference reciprocity norms and boundary maintenance, but these dynamics are interpreted primarily in relation to coordination rather than to reputational considerations or strategic information management. In professions where collaboration and competition coexist, future studies may examine how boundary work and social exchange operate alongside epistemic coordination.

Third, the study draws on interviews with members of a single focal LINE group situated within one national context and one professional community. This design does not support cross-system comparison, estimates of the prevalence of the reconstructed mechanisms, or claims that the contextual features of this case are necessary or sufficient for the mechanisms to occur elsewhere. The scope of analytic generalization is instead at the level of the mechanism-based account developed from the focal case, which can serve as a conceptual lens for comparison with other settings. Future research may pursue comparative designs across media systems, professional cultures, or platform environments, and may examine whether and how the mechanisms occur or vary in other professions characterized by time-critical coordination and distributed expertise.

Fourth, the first author’s insider position provided contextual familiarity but may also have influenced which patterns received analytical attention and how they were interpreted, even though the first author documented insider assumptions in analytic memos, examined negative cases, and maintained an audit trail.

Finally, the study did not collect or analyze LINE message histories, screenshots, timestamps, message sequences, or verbatim interactional transcripts. Consequently, it cannot establish the turn-by-turn sequencing, response timing, or microdynamics of particular exchanges. The three mechanisms are therefore analytic reconstructions rather than findings from direct digital interaction analysis. Future research using message-level data obtained with consent from the group members whose communications are analyzed could examine these interactional dynamics directly. Quantitative or mixed-methods studies could also operationalize these mechanisms to examine whether, under what conditions, and how they recur or vary across professional settings.

By reconstructing three interrelated mechanisms from recurrent patterns in participants’ accounts of how distributed expertise became actionable, this study contributes an interview-grounded account of knowledge coordination in a high-pressure occupational setting.

## Supporting information

S1 Appendix Anonymized semi-structured interview guide in English translation. (DOCX)

S2 Appendix Theme development and participant coverage table. (DOCX)
