# Supplementary material for: Knowledge coordination in a semi-closed IM network under time-critical conditions: Making distributed expertise actionable
Source: PLoS One. 2026 Sep 30;21(9):e0359728. doi: 10.1371/journal.pone.0359728 (PMC13626319; doi:10.1371/journal.pone.0359728)
Supplement: Supplementary file 1 [file pone.0359728.s001.docx]

**S1 Appendix**

**Anonymized Semi-Structured Interview Guide**

*English Translation*

Study: Knowledge Coordination in a Semi-Closed IM Network Under Time-Critical Conditions: Making Distributed Expertise Actionable

Thank you for agreeing to participate. This interview focuses on how journalists use a cross-organizational LINE group in their reporting work, how they coordinate knowledge under time pressure, and how these exchanges relate to professional judgment and practice. You have been informed about the purpose of the study, how the data will be used, your right to withdraw at any time, and the measures taken to protect confidentiality. You have also provided written informed consent. With your permission, I will begin recording and ask you to confirm your consent verbally once the recording starts. Any published materials will use coded identifiers and omit identifying details. Do you have any questions before we begin? May I start recording?

1. Please briefly describe your career as a television political journalist, including how long you have worked in the profession and the reporting roles you have held.

- *Probe: What have been your main work responsibilities and areas of coverage? Have there been any relevant changes in your role?*

2. Please describe how you became a member of the focal cross-organizational LINE group and what role the group plays in your day-to-day reporting work, including the functions it serves.

- *Probe: Could you give a specific example of consulting the group, contributing to it, or coordinating work through it?*

3. How would you describe what you contribute to the group and what you receive from it?
4. When you were learning to work as a television political journalist, how did you learn reporting routines, news judgment, and ways of working with sources?

- *Probe: Who or what helped you learn, and in what situations?*

5. Can you recall a time when something shared or discussed in the focal group helped you learn how to handle a particular reporting task?

- *Probe: What happened before, during, and after the exchange, and what did you take away from it?*

6. When information is lengthy, fragmented, or unfamiliar, how, if at all, do other members help you identify the key points? Please give a specific example.

- *Probe: In that example, what, if anything, did other members do or say that made the key points easier to identify?*

7. Are there aspects of professional judgment or working with sources that are difficult to learn through formal instruction alone? If so, how, if at all, have exchanges in the group helped you understand them?

- *Probe: Could you give a specific example and describe what you learned from it?*

8. How, if at all, is professional experience related to what members contribute to the group and draw from it? Please give examples that informed your view.
9. Please recall a fast-moving reporting situation in which communication within the focal group affected the way work was coordinated. What happened? What did members contribute? What happened next?

- *Probe: What made the situation time-critical, and what happened next?*

10. What kinds of information or materials were exchanged within the group in that situation, and in what form? How, if at all, did they help members continue their reporting work?

- *Probe: What was shared, and how was it used?*

11. Can you describe a situation in which members of the group faced more than one possible explanation or course of action? How did members consider the alternatives?

- *Probe: What differences, if any, emerged, and how did members respond to them?*

12. When a request or developing situation is unclear at first, how, if at all, does the exchange help members arrive at a workable next step?
13. Can you describe a situation in which different members each had only part of the relevant knowledge? How were their contributions brought together?

- *Probe: How did the contributions relate to one another? What happened next?*

14. How, if at all, does coordination through the focal LINE group differ from coordination by phone, face-to-face, or through a newsroom chain of command?

- *Probe: What, if anything, was different about the process?*

15. In group discussions, whose information or interpretation tends to carry greater weight? In a particular situation, what makes that contribution relevant or credible?

- *Probe: What led members to rely on that person or contribution in that situation?*

16. When a group member offers an interpretation or recommendation, what, if anything, helps you understand and assess it? Please give a concrete example.

- *Probe: What, if anything, made the contribution easier to understand or assess?*

17. Can you describe how different members participated as a reporting event unfolded?

- *Probe: At different points, what, if anything, did particular members contribute or seek?*

18. Looking back, has your participation in these exchanges influenced how you plan your reporting, identify clues, compare different interpretations, or make judgments under uncertainty? If so, please describe how.

- *Probe: What, if anything, do you think contributed to those changes?*

19. Thinking about your experience as a whole, how, if at all, has the focal LINE group affected how you obtain the knowledge and background information you need and make reporting decisions under time pressure?
20. Is there anything important about knowledge coordination within the focal group, or about how the group operates, that I have not asked about?

Thank the participant; offer an opportunity to clarify or withdraw any part of the interview responses before the transcript is finalized; reconfirm the confidentiality arrangements and explain the next steps.
