# Supplementary material for: Knowledge coordination in a semi-closed IM network under time-critical conditions: Making distributed expertise actionable
Source: PLoS One. 2026 Sep 30;21(9):e0359728. doi: 10.1371/journal.pone.0359728 (PMC13626319; doi:10.1371/journal.pone.0359728)
Supplement: Supplementary file 2 [file pone.0359728.s002.docx]

**S2 Appendix**

**Theme Development and Participant Coverage Table**

Study: Knowledge Coordination in a Semi-Closed IM Network Under Time-Critical Conditions: Making Distributed Expertise Actionable

| **Higher-order analytic categories** | **Core theme** | **Analytically reconstructed mechanism** | **Contributing N participants (n)** | **Contributing E participants (n)** | **Total (n)** |
| --- | --- | --- | --- | --- | --- |
| Time-critical action orientation; Situational authority and corrective input; Distributed integration of partial expertise | Core theme 1 — Task relevance and situational epistemic authority | Mechanism 1 — Situational redistribution of epistemic authority | 6 | 6 | 12 |
| Externalized professional judgment; Situational authority and corrective input | Core theme 2 — Interpretable cues and externalized professional judgment | Mechanism 2 — Externalization of tacit reasoning into interpretable cues | 4 | 5 | 9 |
| Boundary-spanning access and reciprocal participation; Time-critical action orientation; Distributed integration of partial expertise | Core theme 3 — Partial contributions integrated into actionable judgment | Mechanism 3 — Distributed assembly of interpretive support | 5 | 6 | 11 |

**Note.** N and E refer to the novice and experienced cohorts (n = 6 each). A participant is counted as contributing to a theme if at least one coded segment from that participant’s transcript was assigned to a constituent code of that theme. A participant may contribute to more than one theme. Higher-order categories were analytically overlapping and could contribute to more than one core theme. Participant coverage documents analytic breadth; it does not report the frequency of coded segments, estimate prevalence within the focal group, or test between-cohort differences.
